# Ethnic differences in metabolic syndrome in high-income countries: A systematic review and meta-analysis

**DOI:** 10.1007/s11154-024-09879-9

**Published:** 2024-04-10

**Authors:** Nicholas Kofi Adjei, Florence Samkange-Zeeb, Daniel Boakye, Maham Saleem, Lara Christianson, Mihiretu M. Kebede, Thomas L. Heise, Tilman Brand, Oluwaseun B. Esan, David C. Taylor-Robinson, Charles Agyemang, Hajo Zeeb

**Affiliations:** 1 Department of Public Health, Policy and Systems, University of Liverpool, Waterhouse Building 2nd Floor Block F, Liverpool, L69 3GL UK; 2 Leibniz Institute for Prevention Research and Epidemiology - BIPS, Bremen, Germany; 3 Health Sciences Bremen, University of Bremen, Bremen, Germany; 4 German Cancer Research Center (DKFZ), Heidelberg, Germany; 5 Department of Public Health, Amsterdam Public Health Research Institute, Academic Medical Center, Amsterdam UMC, University of Amsterdam, Amsterdam, The Netherlands

**Keywords:** Metabolic syndrome, Ethnicity, Prevalence, Burden, High-income Countries, Meta-analysis, PROSPERO, CRD42020157189

## Abstract

This review aimed to systematically quantify the differences in Metabolic Syndrome (MetS) prevalence across various ethnic groups in high-income countries by sex, and to evaluate the overall prevalence trends from 1996 to 2022. We conducted a systematic literature review using MEDLINE, Web of Science Core Collection, CINAHL, and the Cochrane Library, focusing on studies about MetS prevalence among ethnic groups in high-income countries. We pooled 23 studies that used NCEP-ATP III criteria and included 147,756 healthy participants aged 18 and above. We calculated pooled prevalence estimates and 95% confidence intervals (CI) using both fixed-effect and random-effect intercept logistic regression models. Data were analysed for 3 periods: 1996–2005, 2006–2009, and 2010–2021. The pooled prevalence of MetS in high-income countries, based on the NCEP-ATP III criteria, was 27.4% over the studied period, showing an increase from 24.2% in 1996–2005 to 31.9% in 2010–2021, with men and women having similar rates. When stratified by ethnicity and sex, ethnic minority women experienced the highest prevalence at 31.7%, while ethnic majority women had the lowest at 22.7%. Notably, MetS was more prevalent in ethnic minority women than men. Among ethnic minorities, women had a higher prevalence of MetS than men, and the difference was highest in Asians (about 15 percentage points). Among women, the prevalence of MetS was highest in Asians (41.2%) and lowest in Blacks/Africans (26.7%). Among men, it was highest in indigenous minority groups (34.3%) and lowest among in Blacks/Africans (19.8%). MetS is increasing at an alarming rate in high-income countries, particularly among ethnic minority women. The burden of MetS could be effectively reduced by tailoring interventions according to ethnic variations and risk profiles.

**Supplementary Information:**

The online version contains supplementary material available at 10.1007/s11154-024-09879-9.

## Background

Metabolic Syndrome (MetS) is a cluster of interrelated metabolic and physiological disorders [1, 2] often linked to insulin resistance [3]. The central components of the syndrome, namely, central obesity, high blood pressure, hyperglycaemia and dyslipidaemia [2–4], have been identified as risk factors for type 2 diabetes [5, 6] and cardiovascular diseases (CVDs), including ischemic heart disease and stroke [3, 7]. Individuals with MetS are two times more likely to suffer from stroke [8] and have a fivefold increased risk of developing type 2 diabetes compared to those without MetS [9].

MetS and its components are a significant public health challenge in high-income countries (HIC), and an emerging public health challenge in Low- and Middle-Income Countries (LMIC) [10, 11]. The prevalence of MetS is increasing to epidemic proportions [12], with a worldwide estimate around 20% to 25% [13]. These figures are expected to rise substantially in the coming years amidst the growing obesity epidemic [14]. MetS has considerable economic impacts [15, 16], for example, MetS costs to the European Union (EU) economy, including productivity loss and informal care, have been estimated to be about €210 billion per year [16].

Despite the increasing prevalence of MetS throughout the world [14], there is some evidence of country [12] and regional variations [17] depending on the definitions used [14]. At present, the two most widely used definitions are those put forward by the International Diabetes Federation (IDF) [18] and the National Cholesterol Education Program Adult Treatment Panel III (NCEP: ATP III) [19]. In Europe, an overall MetS prevalence of 24.3% has been reported when the NCEP:ATP III definition was applied [20]. Australia has a prevalence of 22.1% based on the NCEP:ATP III definition and 30.7% using the IDF definition [21]. In the US, the National Health and Nutrition Examination Survey (NHANES) estimated the prevalence of MetS to be 34.5%, based on the NCEP: ATP III criteria [22].

There are substantial ethnic inequalities in MetS incidence and outcomes. Over the past decades, it has become clearer that the incidence and prognosis of MetS or its components differ by sex, race and ethnicity [23–25]. In some HIC, the prevalence of chronic metabolic disorders, particularly, obesity, type 2 diabetes, hypertension and MetS has been shown to be higher among migrants/ethnic minorities than host/ethnic majority populations [25, 26]. However, this is not a universal finding. For example, some studies from the US report that Hispanic and White groups have a higher prevalence of MetS compared to African Americans [27, 28]. The reasons for these inequalities are complex, and prior findings implicate differences in socioeconomic status (SES) and cultural background [29], differential access to health care and services, and genetic variations as contributing factors to the racial differences in metabolic and cardiovascular diseases [30].

Despite a wealth of studies comparing MetS and its central components among ethnic minority and majority groups [25, 31], the extent of the differences has not been systematically quantified. Therefore, an up-to-date review and overview of the burden of MetS among diverse ethnic groups may be crucial to addressing the inequalities in metabolic diseases. Consequently, the objective of this systematic review and meta-analysis was to quantify the variations of metabolic syndrome among adults of different ethnic groups, with a focus on HIC as classified by the Organization for Economic Co-operation and Development [32].

### Methods

This systematic review followed the updated Preferred Reporting Items for Systematic Reviews and Meta-Analyses (PRISMA) guidelines [33] and the Meta-Analysis of Observational Studies in Epidemiology (MOOSE) guidelines [34]. The protocol was registered in PROSPERO database—(Registration ID: CRD42020157189) [35].

### Search strategy and information sources

The search strategy was developed and conducted by an experienced librarian (LC) in the review team. The search structure combined two concepts using appropriate keywords and controlled vocabulary terms for MetS and racial and ethnic minority groups, including migrants. The search syntax and controlled vocabulary were adapted for subsequent searches in other databases on other platforms. All studies allowing extraction of frequency data on MetS and its core components for different ethnic groups in HIC were included [36]. No limits for language, publication date or study design were applied. The search strategy for all databases is available as supplementary file (supplementary Table 1).

Comprehensive searches were conducted in the following electronic databases in November 2019 and were last updated in January 2023: Medline via Ovid (1946–present); Cumulative Index to Nursing and Allied Health Literature (CINAHL) via Ebsco (1981–present); the Social Science Citation Index (SSCI) (1900–present) and the Science Citation Index (SCI) (1900–present) via Web of Science; and CENTRAL and the Cochrane Database of Systematic Reviews (CDSR) (inception to present) via the Cochrane Library. The references of included studies as well as previously published reviews, studies, and clinical guidelines were hand-searched for additional citations. All results were exported to EndNote reference management software for deduplication. Deduplicated results were imported to an online systematic review management tool, Covidence, for title/abstract and full-text screening.

### Selection criteria

Studies were included if they met the following inclusion criteria: a) adult population (≥ 18 years old) regardless of sex and race/ethnicity in high-income countries [32], b) reported on majority (i.e., White) and minority (i.e., Black, Hispanic, Asian and other) ethnic/racial groups, c) contained observational data that reported prevalence and/or incidence d) primary outcome was MetS, according to accepted diagnostic criteria.

### Screening and selection of studies

In accordance with the study protocol [36], two authors (NKA and FSZ) screened all titles and abstracts from the initial search independently and then compared their findings. The two authors discussed and resolved any arising conflicts. Where no agreement could be reached, a third author (TB) was consulted. NKA and FSZ further independently screened the identified full-texts for eligibility and compared their findings. Similar to the title and abstract process, any arising conflicts were discussed until consensus was reached. TB was consulted where consensus could not be reached. The titles and abstracts identified from the update search were screened by FSZ and HZ independently. The two authors compared their findings and discussed arising conflicts until they reached consensus. NKA was consulted where consensus could not be reached. FSZ and HZ then screened the identified full-texts for eligibility and conflicts were resolved in the same manner as for titles and abstracts.

### Data extraction

NKA and FSZ independently extracted the following data for each study identified during the initial search using an MS Excel data extraction template that was developed a priori: (i) details of the study (first author’s last name, year of publication, country), (ii) methods used in the study (study design and sample characteristics such as sample size, sampling method, ethnic group, age, and sex of participants), (iii) MetS definition criteria, (iv) frequency, incidence, and prevalence of MetS and its components for all adults. Discrepancies in the extracted data were resolved by consensus. Where necessary, HZ was consulted. For the studies identified from the update, FSZ and HZ extracted the respective data independently and resolved any arising discrepancies. NKA was consulted where consensus could not be reached.

### Quality assessment and risk of bias

MS and FSZ assessed the risk of bias of studies identified during the initial search using the National Heart, Lung and Blood Institute’s (NHLBI) Quality Assessment Tool for Observational Cohort and Cross-Sectional Studies [37]. Discrepancies that arose were discussed until consensus was reached. Where consensus could not be reached, NKA and TH were consulted. HZ and FSZ used the same tool to assess the quality of studies identified from the update search. For each stage, the reviewers first conducted the assessment independently, then compared their findings and discussed any discrepancies until consensus was reached. NKA was consulted where consensus could not be reached. An overall risk of bias score was calculated for each study by summing up the score for individual items. The sum score was then categorized to poor, fair and good risk of bias categories.

### Data synthesis and statistical analysis

This study aimed to systematically quantify the variations in the prevalence of MetS among different ethnic groups in HIC by sex, and to assess overall trends in prevalence from 1996 through 2022.

### Narrative synthesis

In conducting summarizing the structured data extracted from individual studies, we first employed a narrative synthesis approach to comprehensively summarize the key attributes and findings reported from each included study. Individual study essential data points such as country, study design, sampling strategy, MetS definition, and the primary outcomes assessed in each study were systematically catalogue and presented in a summary table.

### Quantitative synthesis

Studies using the NCEP-ATP III MetS criteria and providing data for men and women separately were deemed amenable for meta-analysis and were included in the meta-analysis. In brief, we applied the logit transformation method to transform prevalence estimates and calculate their standard errors indirectly [38]. We then used the random-effects models, specifically the random intercept logistic regression model, to calculate summary prevalence estimates and the Hartung-Kanap adjustment to compute the 95% confidence intervals (95% CIs). Where prevalence estimates for different survey periods were presented, the most recent estimates were used for the analysis. Results from the random-effects model are reported as the main results because this model takes into consideration both within and between study heterogeneity [39].

We quantified between-study heterogeneity using Tau-squared (τ^2^) and the I^2^ statistic, where I^2^ > 50% indicates substantial heterogeneity [40]. We employed the Maximum Likelihood (ML) estimator for computing the τ^2^ by utilizing the “metaprop*”* function of the meta r package. Sources of heterogeneity were evaluated statistically using subgroup analysis and random-effects meta-regression, by determining the extent to which age and year of publication explained the observed heterogeneity. Publication bias was first assessed graphically by inspecting symmetry of the funnel plot that displays the individual study effect sizes in the x-axis and their precision (standard error) in the y-axis. We also employed Egger’s test to investigate whether there was evidence of small study effects which may imply potential publication bias. A p-value of less than 0.05 in Egger’s test indicates evidence of small study effects [41].

To determine whether the prevalence of MetS differs by sex and/or ethnicity, we additionally conducted subgroup analyses by combining studies according to sex overall (men and women) and by ethnicity ((majority ethnic women and men (i.e., White) vs. minority ethnic women and men (i.e., Black, Hispanic, Asian and other)). Moreover, among minority women and men, a further analysis was conducted by calculating the prevalence of MetS among African, Hispanic, Asian, and indigenous/other minority descent populations.

All analyses were conducted using the “meta” package (version 6.0–0) [42] in R, version 4.2.0 (R Development Core Team). Statistical tests were two-sided, with a significance level of 5%.

## Results

**Fig. 1:**
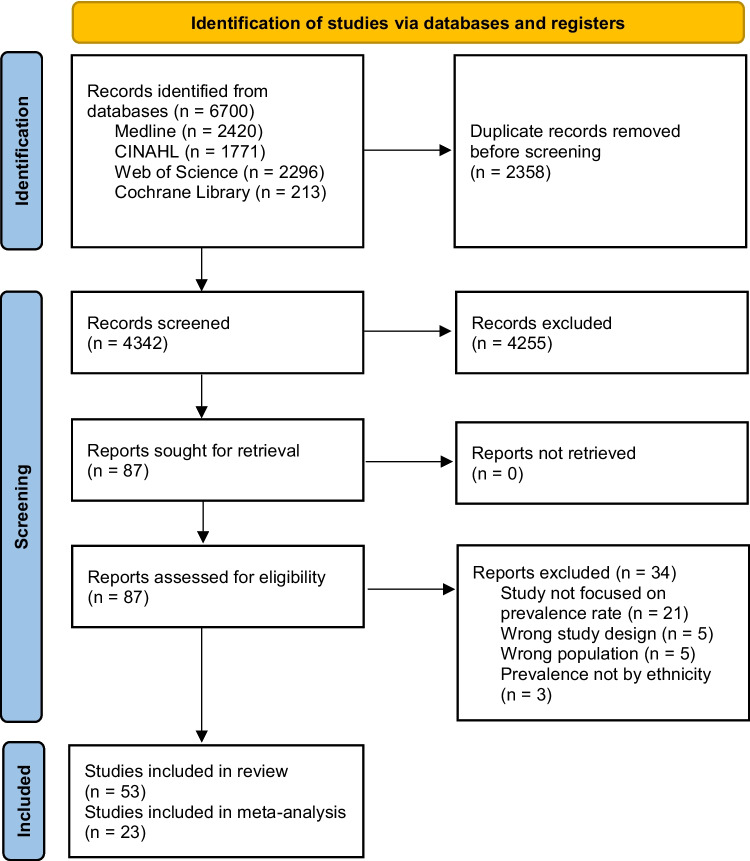
Flow diagram for assessment of eligible studies in the systematic review and meta-analysis

As detailed in the PRISMA flowchart (Fig. 1), a total of 6,700 studies were identified from all searches. After the removal of duplicates and the screening of titles and abstracts, 87 full-texts were reviewed. Of these, 53 met our study inclusion criteria. Reasons for exclusion of the 34 articles after the full-text review have been illustrated in Fig. 1.

### Characteristics of included studies

Almost three-quarters of the included studies were cohort studies and were conducted in the US (38/53) and mostly compared MetS prevalence between Non-Hispanic Whites/White, Non-Hispanic Black/African American and Hispanics/ Mexican American (Table 1). 24 of the 38 studies analysed different periods of cross-sectional data collected within the context of the NHANES [22, 43–65], five used data from The Atherosclerosis in Communities Study (ARIC) [66–70], two from the REasons for Geographic And Racial Differences in Stroke Study (REGARDS) [71, 72], a further two the New York City Health and Nutrition Examination Survey (NYC HANES) [73, 74], and one each from the San Antonio Heart and Framingham Offspring Studies [75], the Kohala Health Research Project [76], The Multi-Ethnic Study of Atherosclerosis (MESA) [77], The Multiethnic Cohort Study (MEC) [78], The Education and Research Towards Health Study (EARTH) [79] and the Northern Manhattan Study (NOMAS) [27]. The remaining 15 studies comprise cross-sectional surveys that were conducted in the Netherlands (n = 4) [80–83], UK (n = 2) [84, 85], Norway (n = 2) [86, 87], New Zealand (n = 2) [88, 89], Canada (n = 2) [90, 91] and one each in Germany [92], Sweden [93] and France [94]. All the studies apart from three [71, 78, 84] applied random sampling methods.

**Table 1:** Characteristics of 53 included study by country

	**Author**	**Country**	**Study Design**	**Sampling Method**	**MetS Criteria**	**MetS prevalence by race/ethnicity (%)**		
						**Total** **(%)**	**Women** **(%)**	**Men** **(%)**
**1.**	Michalsen, 2019	Norway	Prospective cohort	Non-random sample	NCEP:ATP-III	Sami		
							(34.0)	(37.7)
						Non-Sami		
							(39.2)	(38.1)
**2.**	Mcneill, 2004	USA	Cross-sectional	Random sample	NCEP:ATP-III	White		
							(28.2)	(30.6)
						Black		
							(38.4	(25.6)
**3.**	Marcate-Chenard, 2019	USA	Cross-sectional	Random sample	NCEP: ATP-III	Non-Hispanic white		
						(33.8)		
						Black		
						(33.7)		
						Hispanic		
						(32.9)		
**4.**	Loucks, 2007	USA	Cross-sectional	Random sample	AHA/NHLBI	White		
							(28.3)	(31.3)
						Black		
							(29.5)	(19.9)
						Mexican-America		
							(35.0)	(30.1)
**5.**	Liu, 2006	Canada	Cross-sectional	Random sample	NCEP: ATP-III	Oji-Cree		
						(33.3)	(37.2)	(28.2)
						Iniut		
						(13.5)	(18.8)	(6.7)
						Non-Aboriginal Canadian		
						(29.9)	(29.2)	(30.6)
**6.**	Khunti, 2010	UK	Cross-sectional	Non-random sample	NCEP & IDF	White European		
						(34.5)	(31.2)	(38.7)
						South Asian		
						(34.2)	(31.6)	(36.6)
**7.**	Gurka, 2018	USA	Cross-sectional	Random sample	NCEP: ATP-III	Non-Hispanic white		
							(33.2)	(36.2)
						Black		
							(31.9)	(21.7)
						Hispanic		
							(34.4)	(31.9)
**8.**	Gentles, 2007	New Zealand	Cross-sectional	Random sample Random sample	NCEP: ATP-III	White European		
						(16.0)	(15.0)	(17.0)
						Maori		
						(32.0)	(30.0)	(34.0)
						Pacific		
						(39.0)	(37.0)	(41.0)
**9.**	Schumacher, 2008	USA	Cross-sectional		NCEP: ATP-III	White		
							(22.8)	(24.8)
						American Indian and Alaska Native		
							(40.0)	(34.9)
**10.**	Schmidt, 1996	USA	Cross-sectional	Random sample	NCEP: ATP-III	White		
							(4.6)	(10.6)
						African American		
							(4.6)	(11.5)
**11.**	Vernay, 2013	France	Cross-sectional	Random sample	NCEP/ATP III; AHA & NHLBI; IDF; JIS	Born in France		
							(15.8)	(17.5)
						Born outside France		
							(17.0)	(40.2)
**12.**	Chateau-Degat, 2008	Canada	Cross-sectional	Random sample	NCEP ATP-III; IDF; WHO; EGIR	Indian Crees		
						(21.2)	(24.2)	(18.2)
						Iniut		
						(7.7)	(9.9)	(5.7)
						Quebecers		
						(12.5)	10.6)	(14.5)
**13.**	Boden-Albala, 2008	USA	Cross-sectional	Random sample	NCEP: ATP-III	White		
						(39.0)		
						Black		
						(37.0)		
						Hispanic		
						(50.0)		
**14.**	Beydoun, 2008	USA	Cross-sectional	Random sample	NCEP: ATP-III	Non-Hispanic white		
						(26.5)		
						Black		
						(26.5)		
						Mexican American		
						(24.4)		
						Other		
						(27.6)		
**15.**	Tillin, 2005	UK	Cross-sectional	Random sample	NCEP; WHO	European		
							(14.4)	(18.4)
						South Asian		
							(31.8)	(28.8)
						African-Carribeans		
							(23.4)	(15.5)
**16.**	Smiley, 2019	USA	Cross-sectional	Random sample	NCEP: ATP-III	White		
						(15.3)		
						Black		
						(5.6)		
						Hispanic		
						(6.9)		
						Asian		
						(2.2)		
**17.**	Simmons, 2004	New Zealand	Cross-sectional	Random sample	NCEP: ATP-III	White		
							(13.4)	(24.6)
						Maori		
							(51.8)	(52.8)
						Pacific Islander		
							(45.5)	(48.5)
**18.**	Park, 2003	USA	Cross-sectional	Random sample	NCEP: ATP-III	White		
							(22.9)	(24.3)
						Black		
							(20.9)	(13.9)
						Mexican American		
							(27.2)	(20.8)
**19.**	Fruge, 2014	USA	Cross-sectional	Random sample	AHA/NHLBI	Non-Hispanic white		
						(19.7)	(16.8)	(23.2)
						Black		
						(18.2)	(22.1)	(12.9)
						Hispanic		
						(23.8)	(22.1)	(25.4)
**20.**	Salsberry, 2007	USA	Cross-sectional	Random sample	NCEP: ATP-III	White		
							(26.0)	(27.0)
						Black		
							(24.0)	(20.0)
						Mexican American		
							(37.0)	(21.0)
**21.**	Ramphal, 2014	USA	Cross-sectional	Random sample	IDF	Non-Hispanic white		
							(33.4)	(31.6)
						NH-Black		
							(39.5)	(25.0)
						Other		
							(34.6)	(28.9)
						Hispanic/Mexican		
							(40.4)	(37.3)
						American/other		
							(25.9)	(17.4)
**22.**	Mozumdar, 2011	USA	Cross-sectional	Random sample	NCEP: ATP-III	Non-Hispanic white		
							(33.4)	(37.0)
						Black		
							(34.3)	(22.0)
						Mexican American		
							(36.4)	(29.4)
**23.**	Moore, 2017	USA	Cross-sectional	Random sample	JIS	Non-Hispanic/white		
							(25.1)	(24.2)
						Black		
							(20.9)	(16.9)
						Mexican American		
							(18.0)	(15.2)
**24.**	Meigs, 2003	USA	Prospective cohort	Random sample	NCEP:ATP-III; WHO	Framingham Offspring White		
							(21.4)	(26.9)
						Non-Hispanic white		
							(21.3)	(24.7)
						Mexican American		
							(32.8)	(29.0)
**25.**	Mcneill, 2005	USA	Prospective cohort	Random sample	NCEP: ATP-III	White		
							(22.5)	(24.0)
						Black		
							(27.5)	(17.8)
**26.**	Lin, 2007	USA	Cross-sectional	Random sample	NCEP:ATP-III	White		
						(24.1)		
						Black		
						(16.5)		
						Mexican American		
						(29.5)		
**27.**	Keita, 2014	USA	Prospective cohort	Non-random sample	NCEP: ATP-III	White		
						(25.5)		
						Black		
						(26.7)		
**28.**	Jordan, 2012	USA	Cross-sectional	Random sample	NCEP: ATP-III	White		
						(21.8)	(23.5)	(20.1)
						Black		
						(28.5)	(33.4)	(24.0)
						Hispanic		
						(33.9)	(38.2)	(27.4)
						Asian		
						(23.0)	(22.4)	(23.6)
**29.**	Grandinetti, 2005	USA	Cross-sectional	Random sample	NCEP:ATP-III	Caucasian		
						(14.5)		
						Filipino		
						(39.6)		
						Hawaiian		
						(42.0)		
						Japanese		
						(37.0)		
						Other mixed		
						(30.1)		
**30.**	Ford, 2003	USA	Cross-sectional	Random sample	NCEP:ATP-III; WHO	White		
						(24.0)	(22.7)	(25.1)
						African American		
						(21.9)	(26.1)	(16.5)
						Mexican American		
						(32.0)	(36.3)	(28.0)
						Other		
						(20.3)	(19.9)	(20.8)
**31.**	Ford, 2005	USA	Cross-sectional	Random sample	NCEP: ATP-III; IDF	White		
							(33.7)	(36.0)
						African American		
							(33.8)	(21.6)
						Mexican American		
							(37.8)	(32.2)
**32.**	Chichlowska, 2008	USA	Prospective cohort	Random sample	NCEP:ATP-III	White		
							(30.0)	(35.0)
						Black		
							(40.0)	28.0)
**33.**	Chamberlain, 2010	USA	Prospective cohort	Random sample	AHA/NHLBI	White		
						(39.6)		
						Black		
						(45.7)		
**34.**	Akinyemiju, 2017	USA	Prospective cohort	Random sample	JIS	White		
						(38.8)		
						Black		
						(45.8)		
**35.**	Agyemang, 2012	Netherlands	Cross-sectional	Random sample	IDF	White Dutch		
							(26.9)	(33.2)
						African-Surinamese		
							(36.6)	(20.7)
						Hindustani- Surinamese		
							(51.1)	(51.7)
**36.**	Agyemang, 2013	Netherlands	Cross-sectional	Random sample	IDF	White Dutch		
							(20.5)	(29.3)
						Dutch-African		
							(31.4)	(17.7)
						Dutch-Indian		
							(38.4)	(41.6)
						White English		
							(17.8)	(22.5)
						English-African		
							(23.3)	(12.6)
						English-Indian		
							(30.5)	(41.0)
**37.**	Ford, 2010	USA	Cross-sectional	Random sample	JIS	White		
							(31.3)	(38.4)
						African American		
							(38.2)	(25.5)
						Mexican American		
							(41.9)	(34.4)
**38.**	Ford, 2002	USA	Cross-sectional	Random sample	NCEP:ATP-III	White		
						(23.8)	(22.8)	(24.8)
						African American		
						(21.6)	(25.7)	(16.4)
						Mexican American		
						(31.9)	(35.6)	(28.3)
						Other		
						(20.3)	(19.9)	(20.9)
**39.**	Ervin, 2009	USA	Cross-sectional	Random sample	NCEP:ATP-III	Non-Hispanic white		
							(31.5)	(37.2)
						Black		
							(38.8)	(25.3)
						Mexican American		
							(40.6)	(33.2)
**40.**	Campbell, 2016	USA	Cross-sectional	Random sample	NCEP:ATP-III; AHA	Non-Hispanic white		
						(32.6)		
						Black		
						(31.5)		
						Hispanic		
						(34.0)		
						Other		
						(23.0)		
**41.**	Broderstad, 2016	Norway	Cross-sectional	Random sample	IDF	Sami		
							(38.7)	(26.9)
						Non-Sami		
							(39.6)	(30.6)
**42.**	Bindraban, 2008	Netherlands	Cross-sectional	Random sample	NCEP:ATP-III; IDF	White Dutch		
							(16.5)	(17.2)
						African-Surinamese		
							(25.3)	(10.5)
						Hindustani- Surinamese		
							(41.6)	(33.8)
**43.**	Bennet, 2014	Sweden	Cross-sectional	Random sample	JIS	Swedes		
						(40.3)		
						Iraqis		
						(49.2)		
**44.**	Beltran-Sanchez, 2013	USA	Cross-sectional	Random sample	JIS	White		
						(21.8)	(20.3)	(22.9)
						Black		
						(22.7)	(24.5)	(19.0)
						Mexican American		
						(31.9)	(28.5)	(34.8)
**45.**	Agyemang, 2010	Netherland	Cross-sectional	Random sample	IDF	White Dutch		
							(25.8)	(32.5)
						African-Surinamese		
							(35.2)	919.7)
						Hindustani-Surinamese		
							(29.7)	(50.0)
**46.**	Ong, 2019	USA	prospective cohort	Random sample	NCEP:ATP-III	Non-Hispanic white		
						(32.4)		
						African American		
						(37.9)		
						Hispanic American		
						(45.8)		
						Chinese American		
						(29.3)		
**47.**	Lim, 2019	USA	prospective cohort	Non-random	NCEP:ATP-III	White		
							(42.0)	(51.0)
						African-American Latino		
							(19.0)	(21.0)
						Japanese-American		
							(35.0)	(24.0)
						Native Hawaiian		
							(62.0)	(52.0)
						Japanese-American		
							(76.0)	(71.0)
**48.**	Morbach, 2018	Germany	prospective cohort	Random sample	NCEP:ATP-III	Non-migration background (German)		
						(18.5)		
						Migration background		
						(21.0)		
**49.**	Kanchi, 2021	USA	cross-sectional	Random sample	ATP III	Non-Latino White		
						(17.9)	(14.0)	(21.6)
						Non-Latino Black		
						(28.0)	(31.8)	(20.8)
						Latino		
						(28.0)	(31.6)	(23.0)
						Asian		
						(33.8)	(35.9)	(31.1)
**50.**	Okosun, 2019	USA	prospective cohort; cross-sectional analysis	Random sample	NCEP:ATP-III	Non-Hispanic white		
						(31.9)		
						Non-Hispanic Black		
						(25.4)		
						Mexican American		
						(28.7)		
**51.**	Zhu, 2022	USA	cross-sectional analysis (NHANES)	Random sample	IDF 2005	Non-Latino White		
						(25.6)		
						Non-Latino Black		
						(19.3)		
						Latino		
						(31.4)		
						Asian American		
						(22.8)		
**52.**	Ghosh, 2021	USA	cross-sectional analysis (NHANES)	Random sample	NCEP:ATP-III	Non-Latino White		
							(22.2)	(21.8)
						Non-Latino Black		
							(23.6)	(18.0)
						Mexican/Hispanic		
							(18.4)	(18.9)
**53.**	Carabello, 2022	USA	cross-sectional analysis (NHANES)	Random sample	Harmonized definition IDF, NHLB, AHA, WHF, IAS, IASO	Non Hispanic White		
						(42.9)		
						Foreign Born Mexican		
						< 10y (43)		
						10 + y (50.7)		
						US Born Mexican		
						(50.4)		

### Participants

The sample sizes of the included studies ranged from 969 [74] to 33,035 participants [55], and the participants were aged 18 and above. Thirty-seven of the studies reported prevalence data for men and women separately (Supplementary Table 2).

### Definition of MetS

In more than 70% of the studies included in the review (n = 37) [27, 43–45, 47–49, 51, 55, 57–60, 62, 64, 66–69, 71, 73, 75–79, 83–86, 88–92, 94, 95], MetS was defined based on the US NCEP-ATP III guidelines, with 9 of them using a combination of the NCEP-ATP III and other guidelines such as those from the WHO or the IDF [22, 43, 55, 75, 83–85, 91, 94]. The current NCEP-ATP III criteria defines MetS as the presence of ≥ 3 of the following components: 1) waist circumference ≥ 102 cm in men and ≥ 88 cm in women; 2) TG level ≥ 150 mg/dL; 3) HDL-C level < 40 mg/dL in men and < 50 mg/dL in women; 4) blood pressure ≥ 130/85 mm Hg or taking hypertension medications; and 5) fasting glucose level ≥ 100 mg/dL or taking diabetes medications. The rest of the studies applied the Joint Interim Statement (JIS) criteria (n = 5) [49, 51, 55, 71, 92], the IDF (n = 5) [54, 61, 81, 82, 87] and the American Heart Association/National Heart. Lung, and Blood Institute (AHA/NHLBI) criteria (n = 3) [46, 53, 70].

### Risk of bias assessment

Based on the NHLBI tool, the methodological quality of 7 of the studies [37] were rated “good” and 9 were “poor”. The rest were rated as “fair” (Fig. 6 Supplementary pp 11).

### Meta-analysis

Among the 37 studies that used the NCEP-ATP III MetS criteria, 23 [44, 47, 49, 51, 57, 62, 64, 66, 68, 68, 69, 73–75, 78, 79, 83, 85, 86, 89–91, 95] provided data for men and women separately and were included in the meta-analysis (Table 2). 19 (82%) of the studies were from North America, and 4 (18%) from Europe/Oceania (three from Europe and one from New Zealand). The sample size of the individual studies included in the meta-analysis ranged from 920 to 14,502 participants and the combined sample comprised 147,756 aged 18 years or older.

**Table 2:** Characteristics of 23 studies that reported the prevalence of metabolic syndrome by sex using the NCEP-ATP III criteria

**No.**	**First author's name and year of publication**	**Country**	**Sample size (N)**	**Age groups**	**Racial/Ethnic group comparison**	**Women**	**Men**
**1.**	Michalsen, 2019	Norway	5,866	40–79	Sami, Non-Sami	(39.2)/(34.0)	(38.1)/(37.7)
**2.**	McNeill, 2004	USA	14,502	45–64	White, Black	(28.2)/(38.4)	(30.6)/25.6)
**3.**	Liu, 2006	Canada	3,476	≥ 18	Oji-Cree Indians, Iniut, Non-Aboriginal Canadians	(37.2)/(18.8)/(29.2)	(28.2)/(6.7)/(30.6)
**4.**	Gurka, 2018	USA	3,820	20–65	Non-Hispanic white, non-Hispanic Black, Hispanic	(33.2)/(31.9)/(34.4)	(36.2)/(21.7)/(31.9)
**5.**	Schumacher, 2008	USA	11,631	≥ 20	White, American Indian/Alaska native	(22.8)/(40.0)	(24.8)/(34.9)
**6.**	Schmidt, 1996	USA	14,481	45–64	White, African American	(4.6)/(4.6)	(10.6)/(11.5)
**7.**	Chateau-Degat, 2008	Canada	2,613	18–74	Indian Crees, Iniut, Quebecers	(24.2)/(9.9)/(10.6)	(18.2)/(5.7)/(14.5)
**8.**	Tillin, 2005	UK	4,791	40–69	European, South Asian, African-Carribeans	(14.4)/(31.8)/(23.4)	(18.4)/(28.8)/(15.5)
**9.**	Simmons, 2004	New Zealand	2,737	40–79	White European, Maori, Pacific Islander	(19.9)/(50.3)/(45.1)	(23.5)/(56.7)/(46.0)
**10.**	Park, 2003	USA	12,363	≥ 20	White, Black, Mexican American	(22.9)/(20.9)/(27.2)	(24.3)/(13.9)/(20.8)
**11.**	Salsberry, 2007	USA	3,049	≥ 21	NH White, NH Black, Mexican American	(26.0)/(24.0)/(37.0)	(27.0)/(20.0)/(21.0)
**12.**	Mozumdar, 2011	USA	6,962	≥ 20	Non-Hispanic white, NH Black, Mexican American	(31.4)/(36.5)/(42.6)	(36.5)/(24.9)/(36.6)
**13.**	Meigs, 2003	USA	5,961	30–70	White, Non-Hispanic white, Mexican American	(21.4)/(21.3)/(32.8)	(26.9)/(24.7)/(29.0)
**14.**	McNeill, 2005	USA	12,104	45–64	White, Black	(22.5)/(27.5)	(24.0)/(17.8)
**15.**	Jordan, 2012	USA	1,246	≥ 20	NH White, NH Black, Hispanic, NH Asian	(23.5)/(33.4)/(38.2)/(22.4)	(20.1)/(24.0)/(27.4)/(23.6)
**16.**	Ford, 2005	USA	3,349	≥ 20	White, African American, Mexican American	(31.5)/(36.4)/(44.0)	(35.4)/(24.5)/(40.3)
**17.**	Chichlowska, 2008	USA	12,709	45–64	White, Black	(30.0)/(40.0)	(35.0)/(28.0)
**18.**	Ford, 2002	USA	8,814	≥ 20	White, African American, Mexican American, Other	(22.8)/(25.7)/(35.6)/(19.9)	(24.8)/(16.4)/(28.3)/(20.9)
**19.**	Ervin, 2009	USA	3,177	≥ 20	Non-Hispanic white, NH Black, Mexican American	(31.5)/(38.8)/ (40.6)	(37.2)/(25.3)/(33.2)
**20.**	Bindraban, 2008	Netherlands	1,402	35–60	White Dutch, African-Surinamese, Hindustani- Surinamese	(16.5)/(25.3)/(41.6)	(17.2)/(10.5)/(33.8)
**21.**	Lim, 2019	USA	1,794	58–74	White, African-American, Latino, Japanese-American, Native Hawaiian	(42.0)/(19.0)/(35.0)/76.0)/(62.0)	(51.0)/(21.0)/(24.0)/(71.0)/(52.0)
**22.**	Kanchi, 2021	USA	969	≥ 20	Non-Latino White, Non-Latino Black, Latino, Asian	(14.0)/(31.8)/(31.6)/35.9)	(21.6)/(20.8)/(23.0)/(31.1)
**23.**	Ghosh, 2021	USA	10,017	18–80	Non-Latino White, Non-Latino Black, Mex/Hispanic	(22.2)/(23.6)/(18.4)	(21.8)/(18.0)/(18.9)

**No.**	**First author's name and year of publication**	**Total**			**Women**			**Men**		
		**N**	**n (MetS)**	**prev**	**N**	**n (MetS)**	**prev**	**N**	**n (MetS)**	**prev**
**1.**	Michalsen, 2019	5866	2165	36.9	3182	1149	36.1	2684	1016	37.9
**2.**	McNeill, 2004	14502	4404	30.3	7990	2481	31.1	6512	1923	29.5
**3.**	Liu, 2006	3476	1041	29.9	1802	566	31.4	1674	475	28.4
**4.**	Gurka, 2018	3820	1261	33.0	1927	638	33.1	1893	623	32.9
**5.**	Schumacher, 2008	11631	3922	33.7	7055	2497	35.4	4576	1425	31.1
**6.**	Schmidt, 1996	14481	1068	7.3	7981	367	4.6	6500	701	10.8
**7.**	Chateau-Degat, 2008	2613	382	14.6	1365	202	14.8	1248	180	14.4
**8.**	Tillin, 2005	4791	1047	21.8	1175	249	21.2	3616	798	22.1
**9.**	Simmons, 2004	2737	1081	39.4	1494	571	38.2	1243	510	41.0
**10.**	Park, 2003	12363	2731	22.1	6432	1509	23.5	5931	1222	20.6
**11.**	Salsberry, 2007	3049	805	26.4	1486	430	28.9	1563	375	24.0
**12.**	Mozumdar, 2011	6962	2376	34.1	3380	1126	33.3	3582	1250	34.9
**13.**	Meigs, 2003	5961	1535	25.7	3306	817	24.7	2655	718	27.0
**14.**	McNeill, 2005	12104	2816	23.3	6896	1634	23.7	5208	1182	22.7
**15.**	Jordan, 2012	1263	350	27.7	724	224	30.9	539	126	23.3
**16.**	Ford, 2005	3349	1180	35.2	1651	590	35.7	1698	590	34.7
**17.**	Chichlowska, 2008	12709	4197	33.1	7047	2294	32.6	5662	1903	33.6
**18.**	Ford, 2002	8814	2222	25.2	4549	1219	26.8	4265	1003	23.5
**19.**	Ervin, 2009	3177	1093	34.4	1500	525	35.0	1677	568	33.9
**20.**	Bindraban, 2008	1402	328	23.3	823	217	26.4	579	111	19.2
**21.**	Lim, 2019	1794	845	47.1	913	433	47.2	881	412	46.7
**22.**	Kanchi, 2021	920	206	22.3	520	119	22.8	400	87	21.8
**23.**	Ghosh, 2021	10017	2403	23.9	4957	1254	25.3	5060	1147	22.6

**No.**	**First author's name and year of publication**	**Ethnic Majority (women)**			**Ethnic Majority (men)**			**Ethnic Minority (Women)**			**Ethnic Minority (Men)**		
		**N**	**n (MetS)**	**prev**	**N**	**N(MetS)**	**prev**	**N**	**n (MetS)**	**prev**	**N**	**n (Mets)**	**prev**
**1.**	Michalsen, 2019	1899	646	34.0	1571	592	37.7	1283	503	39.2	1113	424	38.1
**2.**	McNeill, 2004	5757	1623	28.2	5124	1568	30.6	2233	857	38.4	1388	355	25.6
**3.**	Liu, 2006	1003	293	29.2	1055	323	30.6	799	273	34.2	619	152	24.6
**4.**	Gurka, 2018	737	245	33.2	737	267	36.2	1190	393	33.0	1156	310	26.8
**5.**	Schumacher, 2008	1887	430	22.8	1712	425	24.8	5168	2067	40.0	2864	1000	34.9
**6.**	Schmidt, 1996	5806	267	4.6	5151	546	10.1	2175	100	10.6	1349	155	11.5
**7.**	Chateau-Degat, 2008	718	76	10.6	699	101	14.5	647	126	19.5	549	79	14.4
**8.**	Tillin, 2005	551	79	14.4	1776	327	18.4	624	170	27.3	1840	471	25.6
**9.**	Simmons, 2004	502	100	19.9	434	102	23.5	992	471	47.5	809	408	50.4
**10.**	Park, 2003	2955	677	22.9	2626	638	24.3	3477	832	23.9	3305	584	17.7
**11.**	Salsberry, 2007	781	203	26.0	839	226	27.0	705	227	32.2	704	149	21.2
**12.**	Mozumdar, 2011	1725	542	31.4	1881	687	36.5	1397	556	39.8	1444	454	31.4
**13.**	Meigs, 2003	2332	498	21.4	1973	520	26.4	974	319	32.8	682	198	29.0
**14.**	McNeill, 2005	5132	1155	22.5	4124	990	24.0	1764	485	27.5	1084	193	17.8
**15.**	Jordan, 2012	191	45	23.5	175	35	20.1	523	179	34.2	357	91	25.6
**16.**	Ford, 2005	892	281	31.5	942	333	35.4	759	309	40.7	756	257	34.0
**17.**	Chichlowska, 2008	5244	1573	30.0	4533	1587	35.0	1803	721	40.0	1129	316	28.0
**18.**	Ford, 2002	1887	430	22.8	1712	425	24.8	2662	789	29.6	2553	577	22.6
**19.**	Ervin, 2009	846	266	31.5	967	360	37.2	654	259	39.6	710	208	29.3
**20.**	Bindraban, 2008	242	40	16.5	244	42	17.2	580	177	30.5	335	69	20.6
**21.**	Lim, 2019	193	69	35.7	207	83	40.0	720	364	50.5	674	329	48.8
**22.**	Kanchi, 2021	198	26	14.0	169	37	21.6	322	93	28.8	231	50	21.6
**23.**	Ghosh, 2021	2367	595	25.1	2503	607	24.2	2590	661	25.5	2557	540	21.1

*prev* prevalence, *NH* Non-Hispanic

### Prevalence of metabolic syndrome

**Fig. 2:**
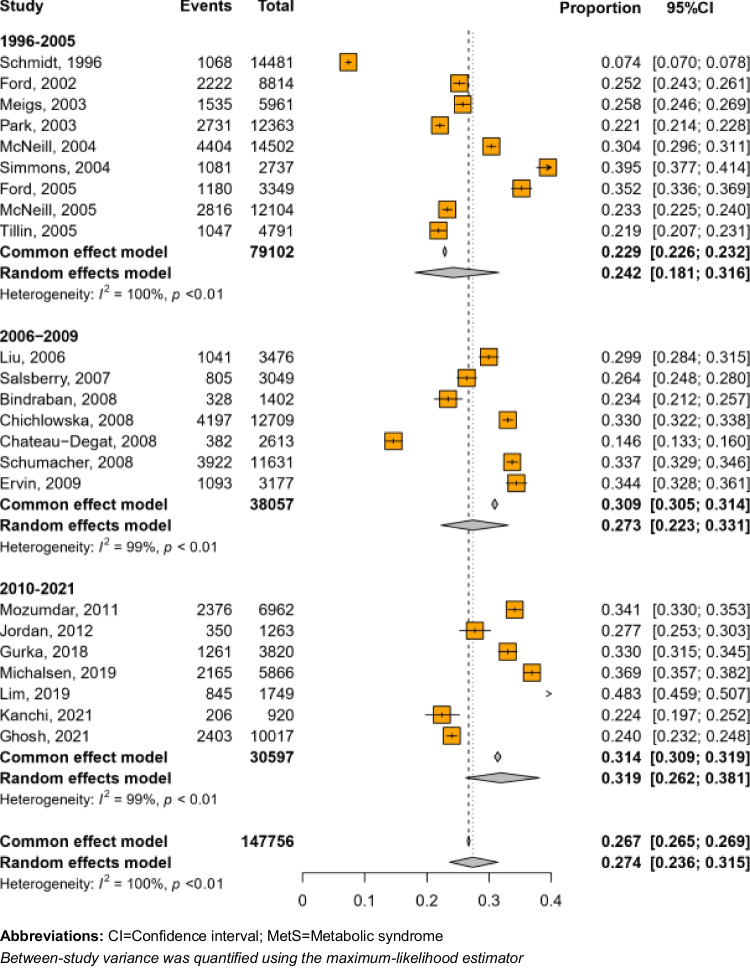
Prevalence of MetS overall and by year of publication

In our meta-analysis of both sexes combined (Fig. 2), the overall prevalence of MetS was 27.4% (95% CI: 23.6% to 31.5%), with evidence of an increase in prevalence over time. For example, in the studies published in 1996–2005, 2006–2009, and 2010–2021, the prevalence of MetS was 24.2%, 27.3%, and 31.9%, respectively. Regarding geographical region, the prevalence of MetS was 26.9% in the studies from North America and 29.8% in those from Europe/Oceania (data not shown). There was a high degree of heterogeneity in all the results (*I*^2^ = 100%, *p* < 0.01), but there was no indication of publication bias (Egger’s test *p* = 0.689). Meta-regression analysis suggested that variations in age of the samples and publication year explained about 17% (*p*_moderation_ = 0.095) and 11% (*p*_moderation_ = 0.252) of the heterogeneity, respectively, and both accounted for about 25% of the heterogeneity. The prevalence of MetS was comparable between women (27.5%, 95%CI: 23.3% to 32.3%; *I*^2^ = 99.2%) and men (26.8%, 95%CI: 23.4% to 30.6%; *I*^2^ = 98.9%) (supplementary Fig. 1).

### Prevalence of metabolic syndrome by ethnicity (majority vs. minority women and men)

**Fig. 3:**
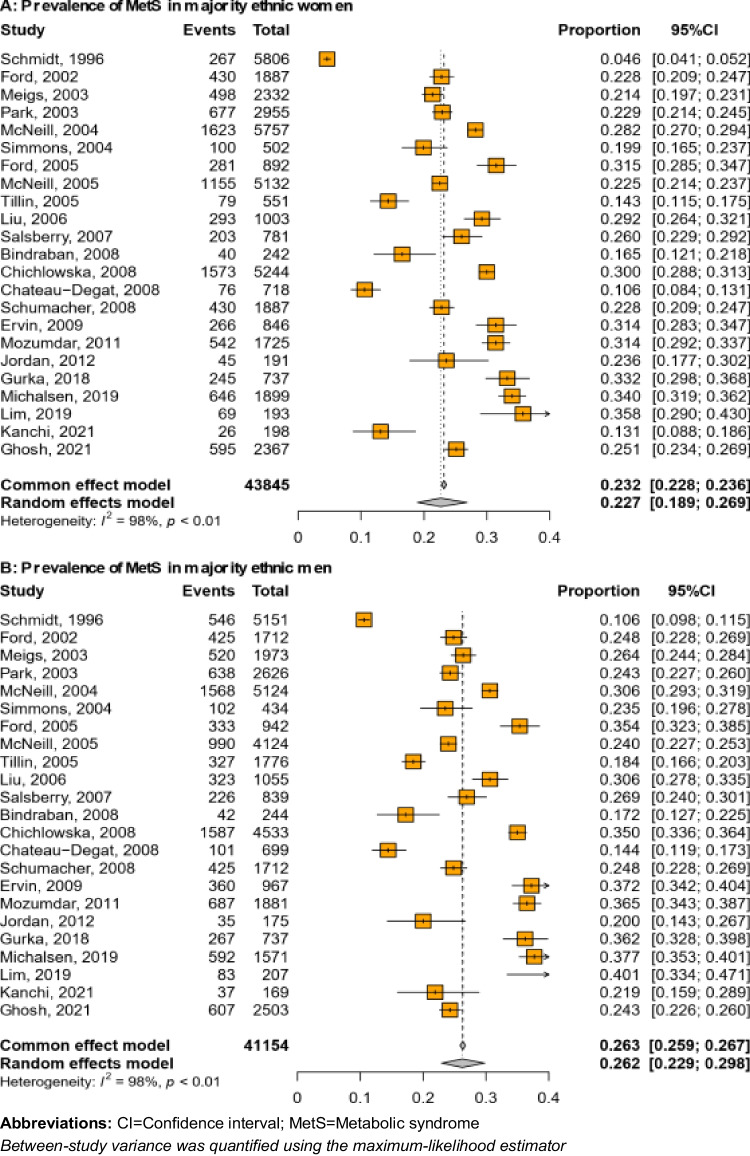
Prevalence of MetS in majority ethnic women (**A**) and men (**B**)

**Fig. 4:**
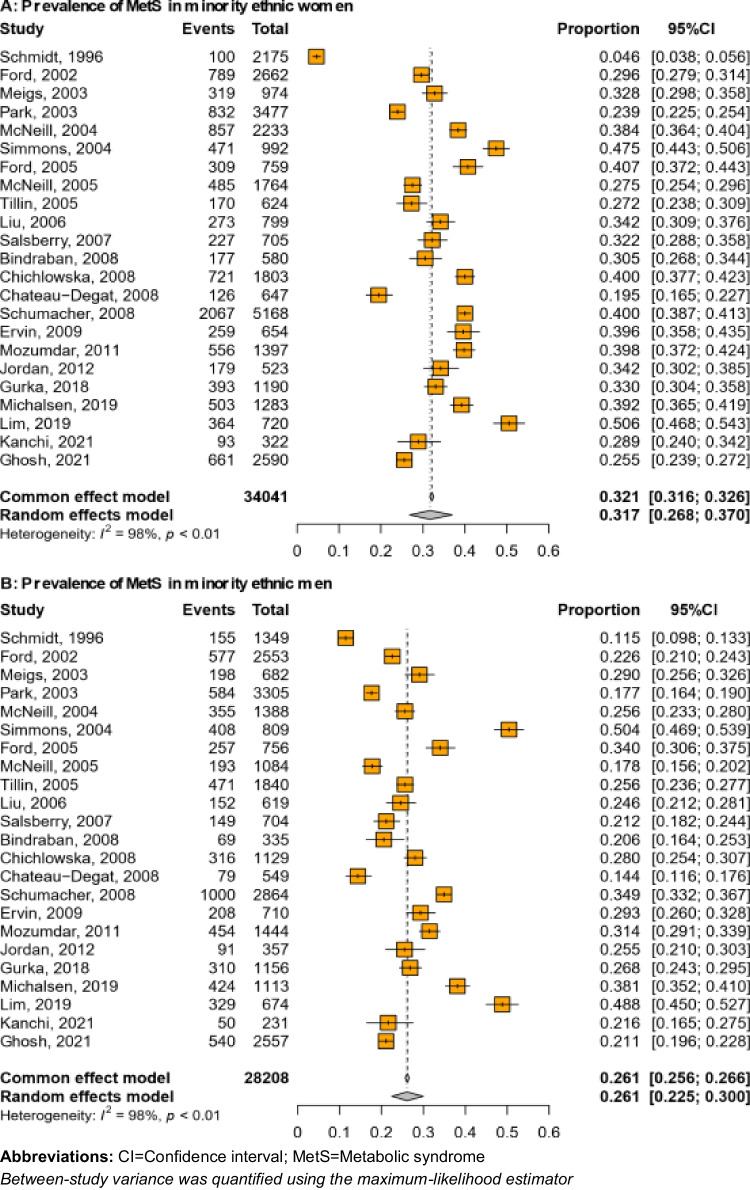
Prevalence of MetS in minority women (**A**) and men (**B**)

In a subgroup analysis of 43,845 and 41,154 ethnic majority women and men respectively (Fig. 3), the prevalence of MetS was 22.7% (95% CI: 18.9% to 26.9%) in women and 26.2% (95% CI: 22.9% to 29.8%) in men. Among the ethnic minority group including 34,041 women and 28,208 men (Fig. 4), the prevalence of MetS was 31.7% (95%CI: 26.8% to 37.0%) in women and 26.1% (95% CI: 22.5% to 30.0%) in men. There was a high degree of heterogeneity in all the results (*I*^2^ > 97%, *p* < 0.01).

Among the ethnic majority women and men, year of publication accounted for 13% and 14% respectively of all the observed heterogeneity, whereas age of the participants accounted for between 3 and 4% of the heterogeneity. In the ethnic minority women, age and year of publication accounted for 14% and 8% of all the observed heterogeneity, respectively, whereas their combination accounted for 20% of the heterogeneity. For men, age explained approximately 40% (*p*_moderation_ < 0.001) of the observed heterogeneity, whereas year of publication explained 7% of the heterogeneity.

### Prevalence of metabolic syndrome among ethnic minorities

Of the studies providing information for ethnic minorities, a further subgroup analysis was conducted by calculating the prevalence of MetS for African (n = 17 studies, supplementary Fig. 2), Hispanic (n = 12 studies, supplementary Fig. 3), Asian (n = 5 studies, supplementary Fig. 4), and indigenous/other minority descent groups (n = 8 studies, supplementary Fig. 5), separately for men and women. Across the minority groups, women had a higher prevalence of MetS than men, and the difference was highest among Asian descent group (about 15 percentage points). Among women, the prevalence of MetS was highest in Asian descent group (41.2%) and lowest in African descent group (26.7%, 95%CI: 21.4%-32.7%). Among men, it was highest in indigenous/other minority groups (34.3%, 95%CI: 30%-38.5%) and lowest in African descent group (19.8%, 95%CI: 17.4%-22.4%).

## Discussion

Although numerous studies on ethnic and sex differences in the prevalence of MetS and its components have been conducted in HIC, a comprehensive and systematic overview of the existing evidence has been lacking. To the best of our knowledge, this is the first systematic review that quantitatively assessed the disparities in MetS among adults of various ethnic origins and sex. We found evidence of sex differences in the prevalence of MetS among minority and majority ethnic/racial groups in HIC. Additionally, the prevalence of MetS appeared to differ among ethnic minority groups – the highest prevalence was observed in Asian descent women and the lowest prevalence in African descent men. We found high heterogeneity across studies which remained unexplained with subgroup analysis and meta-regression analysis. There was no evidence of small-study effect, which may suggest the absence of publication bias.

The overall pooled prevalence of MetS in studies from HIC was 27.4% according to the NCEP-ATP III criteria. The prevalence of MetS was higher (29.8%) in the studies from Europe/Oceania compared to those from North America (26.9%). Without taking ethnicity into account, the prevalence of MetS was similar in women and men. However, when stratified by sex and ethnicity, a sex difference between minority and majority ethnic groups was observed. While the MetS prevalence was lower among women compared to men in the ethnic majority population, men displayed a lower prevalence than women in the ethnic minority population. Overall, we observed the highest MetS prevalence estimates among ethnic minority women, with a large 9 percentage point difference in prevalence between women from the minority ethnic group (31.7%) and those from the majority group (22.7%).

The underlying mechanisms accounting for both ethnic and sex inequalities in MetS and associated cardiometabolic risks remain unclear [96]. However, several potential speculations and explanations have been proposed. These include genetic factors, epigenetic modifications, lifestyle factors (e.g., diet and physical activity), social and environmental determinants, and differences in body composition and fat distribution [1, 97]. Sex-specific differences in body fat distribution, with higher levels of subcutaneous versus visceral fat among women may explain part of the substantial MetS prevalence differences among people of Asian origin living in HIC [98]. Previous studies have suggested that genetic factors may contribute to the higher prevalence of MetS in certain ethnic groups, including polymorphisms in genes involved in lipid metabolism, glucose homeostasis, and inflammation [97]. Similarly, epigenetic modifications, such as DNA methylation, may also play a role in the development of MetS, as these modifications can be influenced by environmental factors and can contribute to changes in gene expression [99, 100].

However, the emergent sex differences across ethnic groups as observed in this current study seem to suggest that dietary patterns, lifestyle and sex-linked biological factors may not explain all cardio-metabolic diseases. Clearly, one cannot underestimate the role of structural risk factors and wider determinants including sociocultural and institutional factors in inequalities in MetS [101]. In the US, systemic racism is debated as a determinant of excess obesity in ethnic minorities [102]. Systemic racism puts ethnic minorities at increased risk for economic hardship including poverty and poor housing conditions [103], chronic stress [104] and an ultra-processed food environment [101]. Recent evidence suggests that ultra-processed foods (i.e., fizzy drinks, sugary cereals, packaged baked goods and ready meals containing food additives, which are often high in calories, sugar and fat) are associated with an increased risk of CVD and death [105, 106]. While both ethnic minority women and men are exposed to these factors, our data surprisingly shows that African decent men exhibit a lower prevalence of MetS compared to ethnic majority men. We speculate that this may be related to other environmental and genetic factors [97]. Nonetheless, it is important to note that most of the causal factors of MetS and its components are preventable and modifiable [107]. Thus, future research studying the causes of MetS can help elucidate the complex interplay of risk factors and how they shape inequalities among diverse population groups across the life course. This may aid in the development of targeted interventions to reduce cardiometabolic risks in ethnic minority women.

The main strength of this current study is the inclusion of several literature search databases which facilitated the identification of numerous studies involving a large number of participants, which enabled deeper investigation through population stratification (i.e., subgroup analysis by sex and ethnicity) to further understand the burden of MetS among diverse racial/ethnic groups in HIC. There are also limitations. First, most of the included studies were carried out in the US. Since countries differ in their ethnic composition, in their history of migration or colonialism, and regarding the socioeconomic disparities across groups, more studies from other countries are needed to confirm the findings of our review. Another limitation is the high degree of between-study heterogeneity, which means that the pooled prevalence estimates should be interpreted with caution. Differences in the mean age of the study populations explained some of the heterogeneity, which is plausible because the risk of MetS is associated with age [108]. However, a sizable extent of the heterogeneity remained unexplained. Even though we suggest interpreting the pooled estimates with caution, we are still convinced that the comparisons across the groups are valid because we included only studies that provided data for all subgroups in the meta-analysis. Hence, it is probable that all subgroups may be affected by this heterogeneity in a similar manner. Second, the choice of a MetS definition obviously affects prevalence estimates, as the use of the IDF definition often leads to higher prevalence estimates as compared to the NCEP – ATP III criteria. Our meta-analysis is based on the latter, and thus the pooled estimates need to be interpreted in light of the definition applied. However, since there was no uniform reporting of MetS according to different definitions across studies, we decided to only compute NCEP – ATP III based pooled prevalence estimates. Third, we used of the year of publication as a proxy measure for the year of study conduct, as the latter was not consistently reported across all studies included in our analysis. Fourth, although we conducted thorough literature searches in multiple established databases for conducting literature reviews, we may have still missed important studies. The current assessment relies on data from 53 studies, including a substantial population of some 80,000 women and men from ethnic minority groups living in HIC. Given the precision of the pooled estimates, large studies with differing results would be required to substantially alter the findings. We find it unlikely that such studies may have been missed, but acknowledge the uncertainty and heterogeneity of findings, as well as the limited study quality of many of the included studies.

Nonetheless, the findings of this systematic review and meta-analysis provide strong evidence that women from ethnic minority groups have an increased prevalence of MetS and can be considered at higher risk of developing MetS. Multiple factors are likely to play a role, but so far, it remains unclear what the main drivers of MetS in this heterogeneous group are. Therefore, more research is needed to identify these factors and to gain an in-depth understanding of what shapes the everyday and health-related behaviours of ethnic minority people, especially women.

In conclusion, the findings of this review have important policy implications for HIC, as MetS has been shown to be an important risk factor for several chronic diseases, including CVDs [1, 2, 12]. Our study shows that this risk factor is unequally distributed across ethnic groups in HIC when taking sex into account. Specifically, women from ethnic minorities display an increased prevalence of MetS. As most of the included studies were from the US, more research is needed to confirm our findings, particularly in the context of other countries. Given that the COVID-19 pandemic has exacerbated existing inequalities and made structural racism a global health concern [109], it is imperative that we understand the driving factors of MetS in women from minority ethnic groups. This understanding is particularly crucial for countries and ethnic groups that are not covered in this review. Improving the awareness, treatment, and control of MetS and its components among ethnic minority populations is crucial in reducing and preventing morbidity and mortality from cardio-metabolic diseases.

### Supplementary Information

Below is the link to the electronic supplementary material.

Supplementary file1 (DOCX 1660 KB)

## Data Availability

All relevant data are within the manuscript and its supporting information files.

## Abbreviations

CDSR: Cochrane Database of Systematic Reviews
CINAHL: Cumulative Index to Nursing and Allied Health Literature
CIs: Confidence Intervals
EU: European Union
HIC: High-income countries
IDF: International Diabetes Federation
LMIC: Low- and Middle-Income Countries
MetS: Metabolic Syndrome
MOOSE: Meta-Analysis of Observational Studies in Epidemiology
NCEP: ATP III: National Cholesterol Education Program Adult Treatment Panel III
NHLBI: National Heart, Lung and Blood Institute’s
NHANES: National Health and Nutrition Examination Survey
PRISMA: Preferred Reporting Items for Systematic Reviews and Meta-Analyses
SES: Socioeconomic status
SSCI: Social Science Citation Index
