# Supplementary material for: Ethnic differences in metabolic syndrome in high-income countries: A systematic review and meta-analysis
Source: Rev Endocr Metab Disord. 2024 Apr 10;25(4):727–50. doi: 10.1007/s11154-024-09879-9 (PMC11294386; doi:10.1007/s11154-024-09879-9)
Supplement: Supplementary file 1 — Supplementary file1 (DOCX 1660 KB) [file 11154_2024_9879_MOESM1_ESM.docx]

**Supplementary appendix:** Protocol for this study

Adjei, N. K., Samkange-Zeeb, F., Kebede, M., Saleem, M., Heise, T. L., & Zeeb, H. (2020). Racial/ethnic differences in the prevalence and incidence of metabolic syndrome in high-income countries: a protocol for a systematic review. *Systematic reviews*, *9*(1), 1-5.

DOI: <https://doi.org/10.1186/s13643-020-01400-y>

**Supplementary Table 1:** Search strategy

MEDLINE(R) and Epub Ahead of Print, In-Process & Other Non-Indexed Citations, Daily and Versions(R) / MEDLINE(R) ALL 1946 – search date via Ovid
Searched/exported 20.11.2019; updated 21.06.2021 and 16.01.2023

| **1.** | ((race* or racial or ethnic* or ethnicit*) adj5 minorit*).ti,ab. |
| --- | --- |
| **2.** | ((raci* or race or ethnic* or minorit* or immigra* or emigra* or migrant* or migration) adj5 (group* or communit* or population*)).ti,ab. |
| **3.** | exp minority groups/ |
| **4.** | exp minority health/ |
| **5.** | exp ethnic groups/ |
| **6.** | exp "emigrants and immigrants"/ |
| **7.** | or/1-6 |
| **8.** | METS.ti,ab. |
| **9.** | metabolic syndrome.ti,ab. |
| **10.** | exp metabolic syndrome/ |
| **11.** | or/8-10 |
| **12.** | 7 and 11 |
| **13.** | ((race* or racial or ethnic* or raci* or immigra* or emigra* or migrant* or migration or minorit*) adj15 (METS or metabolic syndrome)).ti,ab. |
| **14.** | 12 or 13 |

Cumulative Index to Nursing and Allied Health Literature -CINAHL via EBSCO 1981-present
Searched/exported 20.11.2019; updated 21.06.2021 and 16.01.2023

| **1.** | MH "Ethnic Groups+" |
| --- | --- |
| **2.** | MH "Minority Groups+" |
| **3.** | MH "Emigration and Immigration+" |
| **4.** | TI (((raci* or race or ethnic*or minorit* or immigra* or emigra* or migrant* or migration) n5 (group* or communit* or population*))) OR AB (((raci* or race or ethnic* or minorit* or immigra* or emigra* or migrant* or migration) n5 (group* or communit* or population*))) |
| **5.** | TI (((race* or racial or ethnic* or ethnicit*) n5 minorit*)) OR AB (((race* or racial or ethnic* or ethnicit*) n5 minorit*)) |
| **6.** | S1 OR S2 OR S3 OR S4 OR S5 |
| **7.** | MH "Metabolic Syndrome X+" |
| **8.** | TI ("metabolic syndrome" OR "mets") OR AB ("metabolic syndrome" OR "mets") |
| **9.** | S7 OR S8 |
| **10.** | S6 AND S9 |
| **11.** | TI (((race* or racial or ethnic* or raci* or immigra* or emigra* or migrant* or migration or minorit*) n15 ("mets" or "metabolic syndrome"))) OR AB (((race* or racial or ethnic* or raci* or immigra* or emigra* or migrant* or migration or minorit*) n15 ("mets" or "metabolic syndrome"))) |
| **12.** | S10 OR S11 |

Social Science Citation Index & Science Citation Index via Web of Science Platform from 1900 to present
Searched/exported 20.11.2019; updated 21.06.2021 and 16.01.2023

| **1.** | TS=((race* or racial or ethnic* or ethnicit*) near/5 minorit*) |
| --- | --- |
| **2.** | TS=((raci* or race or ethnic* or minorit* or immigra* or emigra* or migrant* or migration) near/5 (group* or communit* or population*)) |
| **3.** | TS="minority health" |
| **4.** | TS=("metabolic syndrome" OR "METS") |
| **5.** | #1 OR #2 OR #3 |
| **6.** | #4 AND #5 |
| **7.** | TS=((race* or racial or ethnic* or raci* or immigra* or emigra* or migrant* or migration or minorit*) near/15 (METS or "metabolic syndrome")) |
| **8.** | #7 or #6 |

CENTRAL and Cochrane Database of Systematic Reviews via the Cochrane Library from inception to present
Searched/exported 20.11.2019; updated 21.06.2021 and 16.01.2023

| **1.** | ((race* or racial or ethnic* or ethnicit*) near/5 minorit*):ti,ab |
| --- | --- |
| **2.** | ((raci* or race or ethnic* or minorit* or immigra* or emigra* or migrant* or migration) near/5 (group* or communit* or population*)):ti,ab |
| **3.** | MeSH descriptor: [Minority Groups] explode all trees |
| **4.** | MeSH descriptor: [Minority Health] explode all trees |
| **5.** | MeSH descriptor: [Ethnic Groups] explode all trees |
| **6.** | MeSH descriptor: [Emigrants and Immigrants] explode all trees |
| **7.** | {OR #1-#6} |
| **8.** | METS:ti,ab |
| **9.** | "metabolic syndrome":ti,ab |
| **10.** | MeSH descriptor: [Metabolic Syndrome] explode all trees |
| **11.** | {OR #8-#10} |
| **12.** | #7 AND #11 |
| **13.** | ((race* or racial or ethnic* or raci* or immigra* or emigra* or migrant* or migration or minorit*) near/15 (METS or "metabolic syndrome")):ti,ab |
| **14.** | #12 OR #13 |

**Supplementary Table 2:** Characteristics of 37 studies that reported the prevalence of metabolic syndrome by sex

| **No.** | **Author** | **Racial/Ethnic group comparison** | **Women** | **Men** |
| --- | --- | --- | --- | --- |
| **1.** | Michalsen, 2019 | Sami/Non-sami | (39.2)/(34.0) | (38.1)/(37.7) |
| **2.** | McNeill, 2004 | White/Black | (28.2)/(38.4 | (30.6)/25.6) |
| **3.** | Loucks, 2007 | White /Black/Mexican-America | (28.3)/(29.5)/(35.0) | (31.3)/(19.9)/(30.1) |
| **4.** | Liu, 2006 | Oji-Cree /Iniut / Non-Aboriginal Canadian | (37.2)/(18.8)/(29.2) | (28.2)/(6.7)/(30.6) |
| **5.** | Khunti, 2010 | White European/ South Asian | (31.2)/(31.6) | (38.7)/(36.6) |
| **6.** | Gurka, 2018 | Non-Hispanic white/Black/Hispanic | (33.2)/(31.9)/(34.4) | (36.2)/(21.7)/(31.9) |
| **7.** | Gentles, 2007 | White European (16.0)/Maori (32.0)/ Pacific (39.0) | (15.0)/(30.0)/(37.0) | (17.0)/(34.0)/(41.0) |
| **8.** | Schumacher, 2008 | White/American Indian and Alaska Native | (22.8)/(40.0) | (24.8)/(34.9) |
| **9.** | Schmidt, 1996 | White/African American | (4.6)/(4.6) | (10.6)/(11.5) |
| **10.** | Vernay, 2013 | born in France,/born outside France | (15.8)/(17.0) | (17.5)/(40.2)/ |
| **11.** | Chateau-Degat, 2008 | Indian Crees/Iniut /Quebecers | (24.2)/(9.9)/(10.6) | (18.2)/(5.7)/(14.5) |
| **12.** | Tillin, 2005 | European /South Asian /African-Carribeans | (14.4)/(31.8)/(23.4) | (18.4)/(28.8)/(15.5) |
| **13.** | Simmons, 2004 | White European/ Maori/Pacific | (13.4)/(51.8)/(45.5) | (24.6)/(52.8)/(48.5) |
| **14.** | Park, 2003 | White /Black/Mexican American | (22.9)/(20.9)/(27.2) | (24.3)/(13.9)/(20.8) |
| **15.** | Fruge, 2014 | Non-Hispanic white//Black /Hispanic | (16.8)/(22.1)/(22.1) | (23.2)/(12.9)/(25.4) |
| **16.** | Salsberry, 2007 | White /Black/Mexican American | (26.0)/(24.0)/(37.0) | (27.0)/(20.0)/(21.0) |
| **17.** | Ramphal, 2014 | Non-Hispanic white/NH-Black/Other Hispanic/Mexican American/other | (33.4)/(39.5)/(34.6)/(40.4) /(25.9) | (31.6)/(25.0)/(28.9)/(37.3) /(17.4) |
| **18.** | Mozumdar, 2011 | Non-Hispanic white/ Black/ Mexican American | (33.4)/(34.3)/(36.4) | (37.0)/(22.0)/(29.4) |
| **19.** | Moore, 2017 | Non-Hispanic white/Black/Mexican American | (25.1)/(20.9)/(18.0) | (24.2)/(16.9)/(15.2) |
| **20.** | Meigs, 2003 | Framingham Offspring White/Non-Hispanic white/Mexican American | (21.4)/(21.3)/(32.8) | (26.9)/(24.7)/(29.0) |
| **21.** | McNeill, 2005 | White/Black | (22.5)/(27.5) | (24.0)/(17.8) |
| **22.** | Jordan, 2012 | White / Black/ Hispanic /Asian | (23.5)/(33.4)/(38.2)/(22.4) | (20.1)/(24.0)/(27.4)/(23.6) |
| **23.** | Ford, 2003 | White /African American / Mexican American/ Other | (22.7)/(26.1)/(36.3)/(19.9) | (25.1)/(16.5)/(28.0)/(20.8) |
| **24.** | Ford, 2005 | White /African American / Mexican American | (33.7)/(33.8)/(37.8) | (36.0)/(21.6)/(32.2) |
| **25.** | Chichlowska, 2008 | White/Black | (30.0)/(40.0) | (35.0)/(28.0) |
| **26.** | Agyemang, 2012 | White Dutch/ African-Surinamese/ Hindustani- Surinamese | (26.9)/(36.6)/(51.1) | (33.2)/(20.7)/(51.7) |
| **27.** | Agyemang, 2013 | White Dutch/ Dutch-African/Dutch-Indian/White English/ English-African/ English-Indian | (20.5)/(31.4)/(38.4)/(17.8)/23.3)/30.5) | (29.3)/(17.7)/(41.6)/(22.5)/12.6)/41.0) |
| **28.** | Ford, 2010 | White/African American/Mexican American | (31.3)/(38.2)/(41.9) | (38.4)/(25.5)/(34.4) |
| **29.** | Ford, 2002 | White/African American /Mexican American/Other | (22.8)/(25.7)/(35.6)/(19.9) | (24.8)/(16.4)/(28.3)/(20.9) |
| **30.** | Ervin, 2009 | Non-Hispanic white/ Black/Mexican American | (31.5)/(38.8)/(40.6) | (37.2)/(25.3)/(33.2) |
| **31.** | Broderstad, 2016 | Sami/ Non-sami | (38.7)/(39.6) | (26.9)/(30.6) |
| **32.** | Bindraban, 2008 | White Dutch/African-Surinamese/ Hindustani- Surinamese | (16.5)/(25.3)/(41.6) | (17.2)/(10.5)/(33.8) |
| **33.** | Beltran-Sanchez, 2013 | White /Black/Mexican American | (20.3)/(24.5)/(28.5) | (22.9)/(19.0)/(34.8) |
| **34.** | Agyemang, 2010 | White Dutch/African-Surinamese/Hindustani- Surinamese | (25.8)/(35.2)/(29.7) | (32.5)/(19.7)/(50.0) |
| **35.** | Lim, 2019 | White, African-American, Latino, Japanese-American, Native Hawaiian, Japanese-American | (42.0)/(19.0)/(35.0)/(62.0)/76.0) | (51.0)/(21.0)/(24.0)/(52.0)/(71.0) |
| **36.** | Kanchi, 2021 | Non-Latino White /Non-Latino Black/Latino/Asian | (14.0)/(31.8)/(31.6)/35.9) | (21.6)/(20.8)/(23.0)/(31.1) |
| **37.** | Ghosh, 2021 | Non-Latino White/ Black/ Hispanic | (22.2)/(23.6)/ (18.4) | (21.8)/ (18.0)/(18.9) |

**Supplementary Figure 1:** Prevalence of MetS in women (A) and men (B)


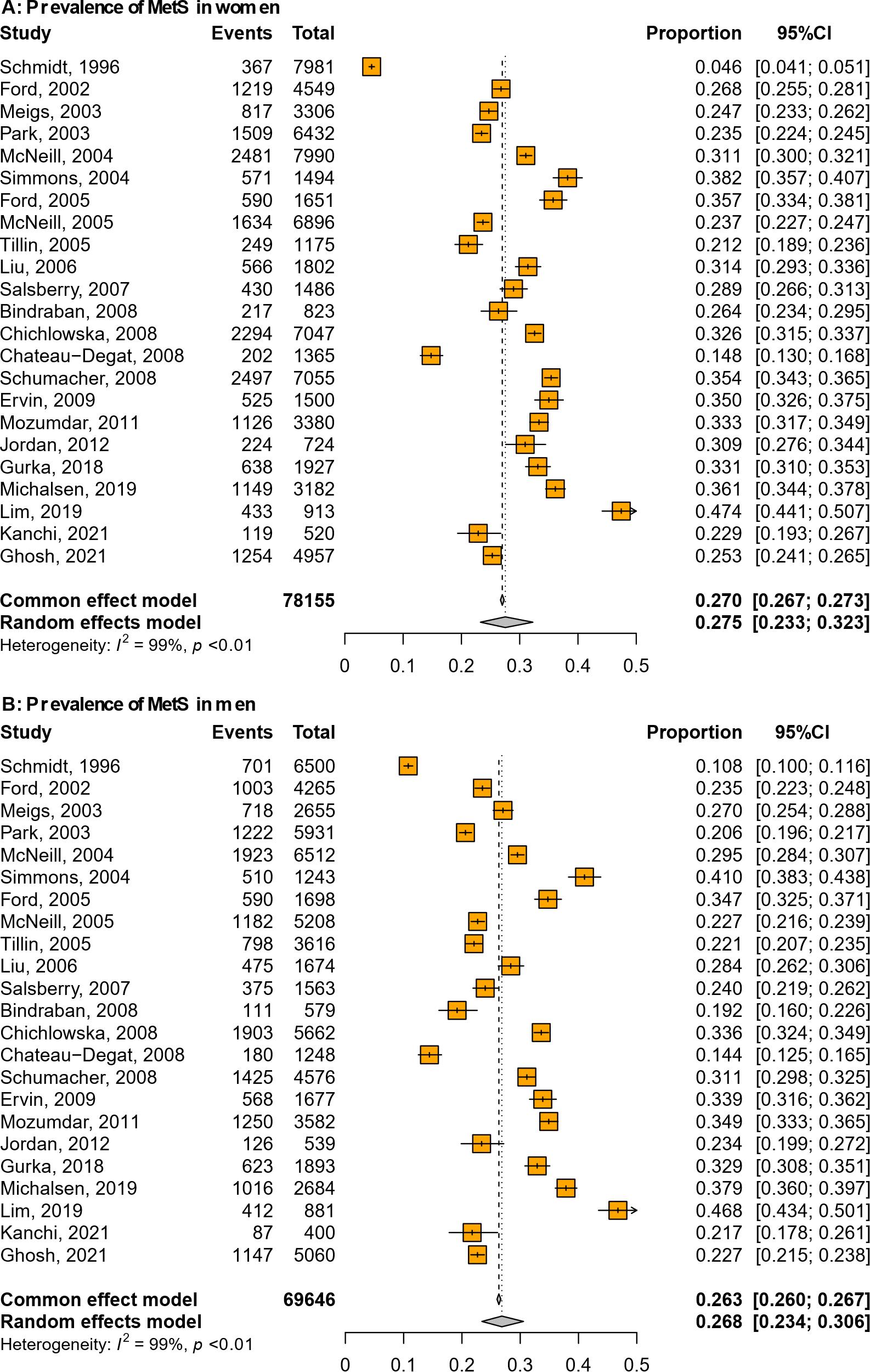


**Abbreviations:** CI=Confidence interval; MetS=Metabolic syndrome

*Between-study variance was quantified using the maximum-likelihood estimator*

**Supplementary Figure 2:** Prevalence of MetS in Black/African women (A) and men (B)


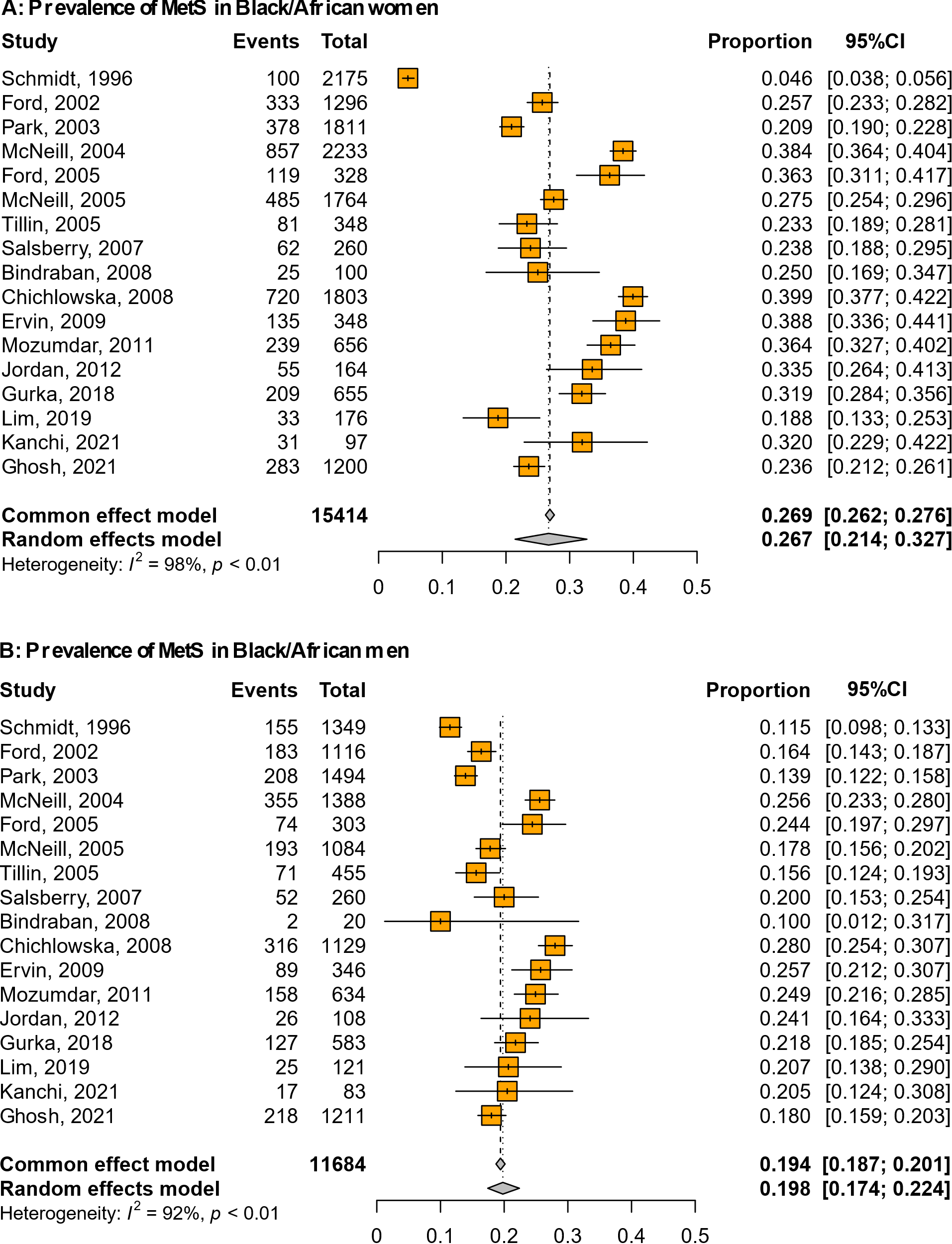


**Abbreviations:** CI=Confidence interval; MetS=Metabolic syndrome

*Between-study variance was quantified using the maximum-likelihood estimator*

**Supplementary Figure 3:** Prevalence of MetS in Hispanic women (A) and men (B)


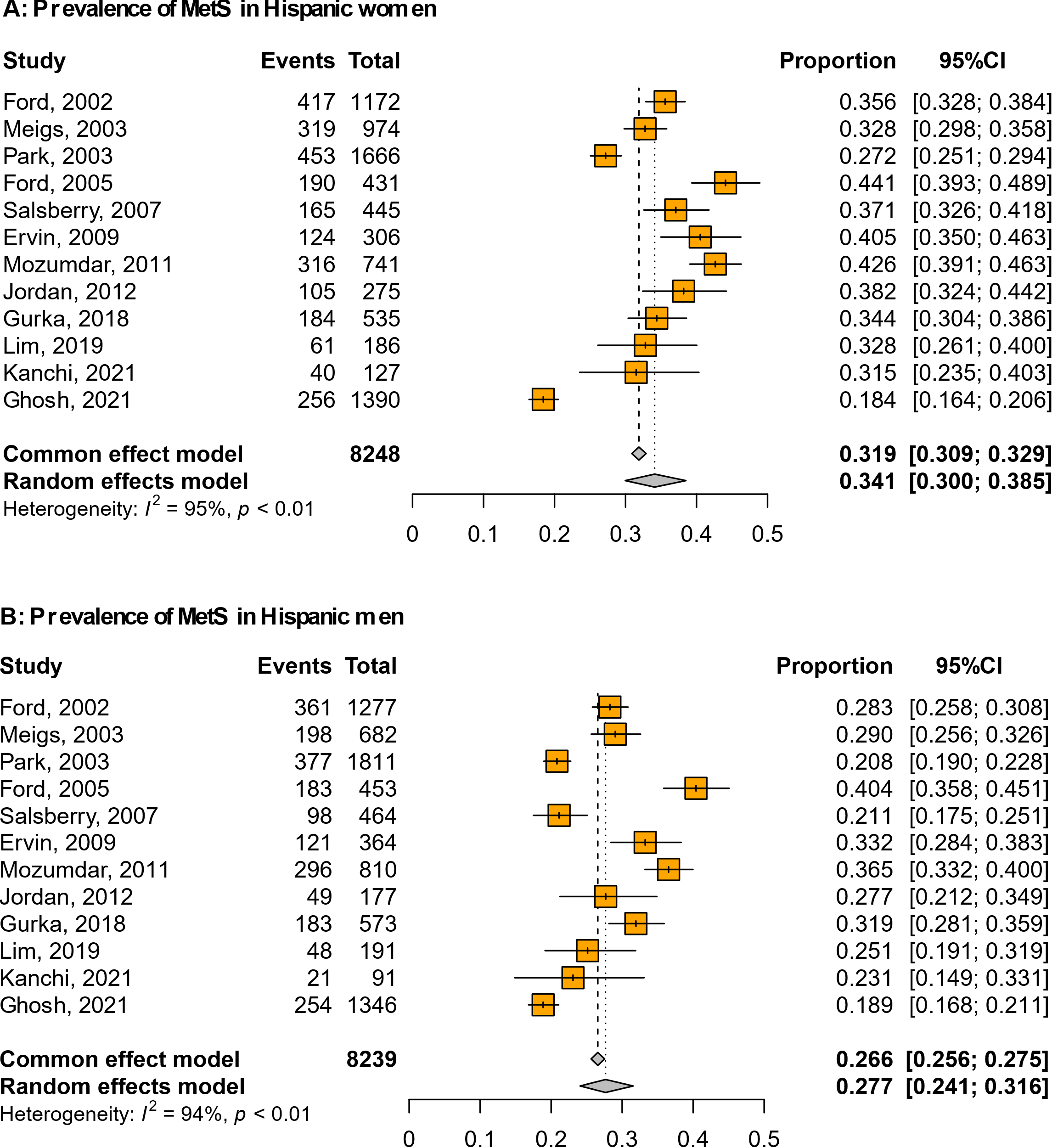


**Abbreviations:** CI=Confidence interval; MetS=Metabolic syndrome

*Between-study variance was quantified using the maximum-likelihood estimator*

**Supplementary Figure 4:** Prevalence of MetS in Asian women (A) and men (B)


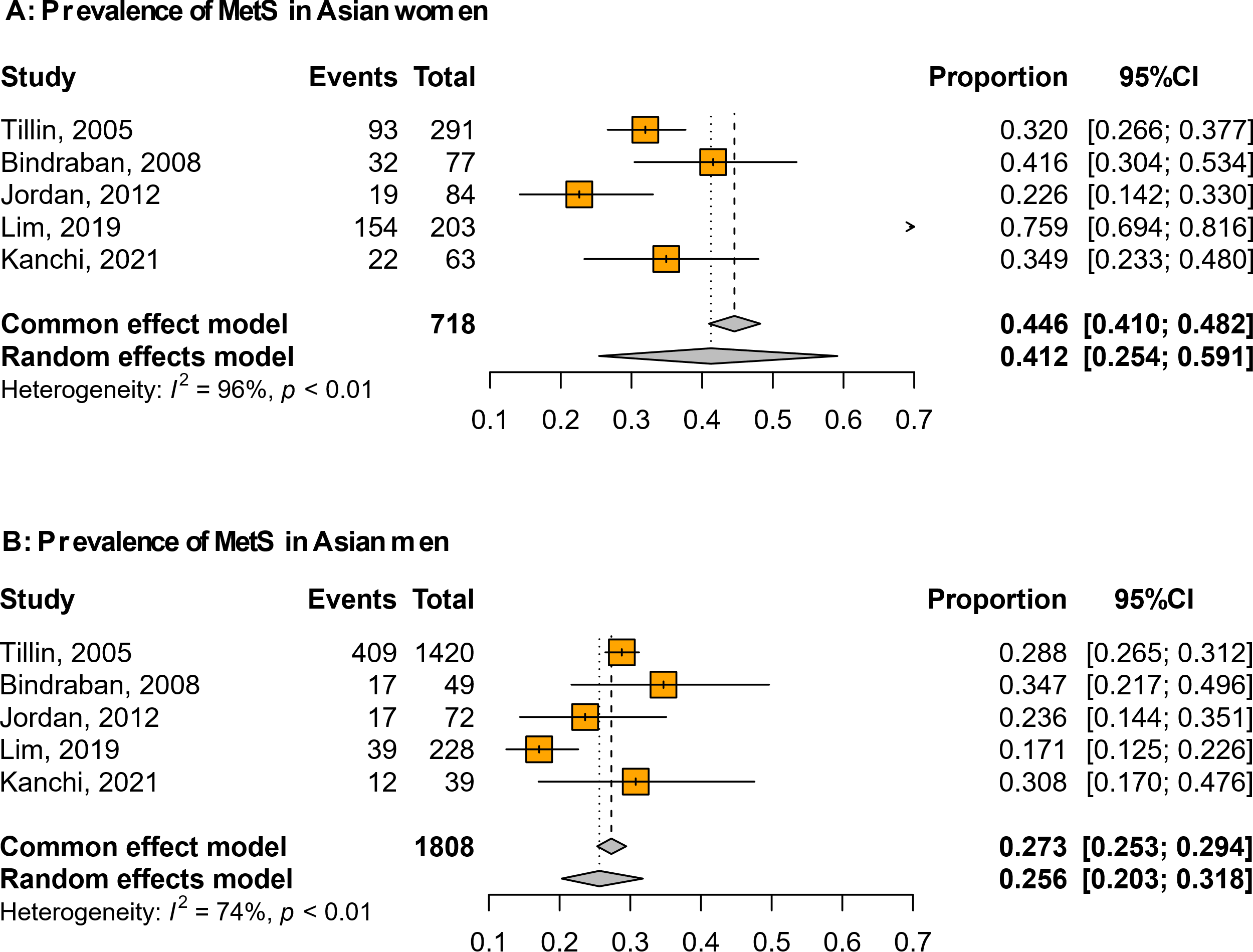


**Abbreviations:** CI=Confidence interval; MetS=Metabolic syndrome

*Between-study variance was quantified using the maximum-likelihood estimator*

**Supplementary Figure 5:** Prevalence of MetS in Indigenous/Other minority women (A) and men (B)


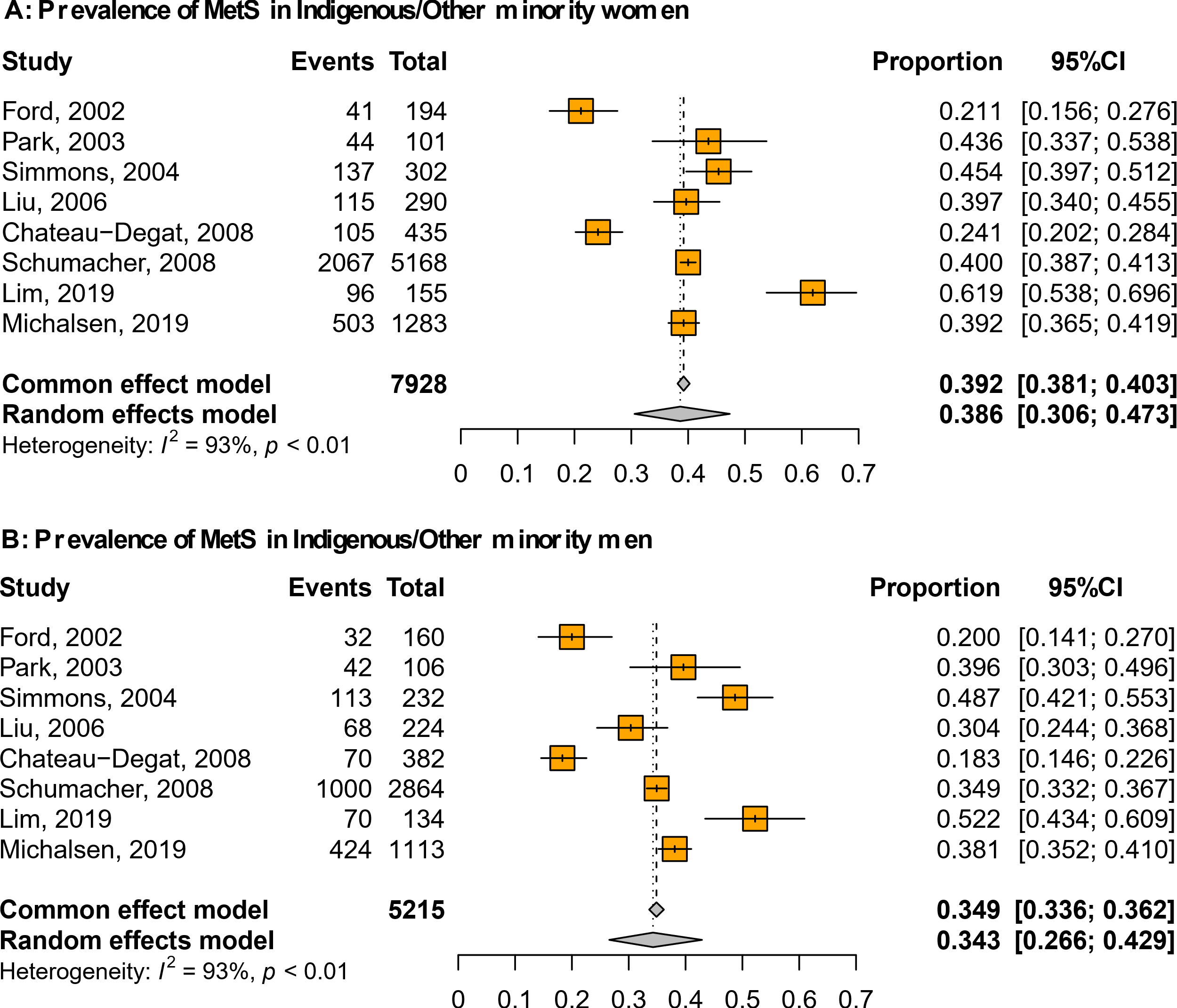


**Abbreviations:** CI=Confidence interval; MetS=Metabolic syndrome

*Between-study variance was quantified using the maximum-likelihood estimator*

*
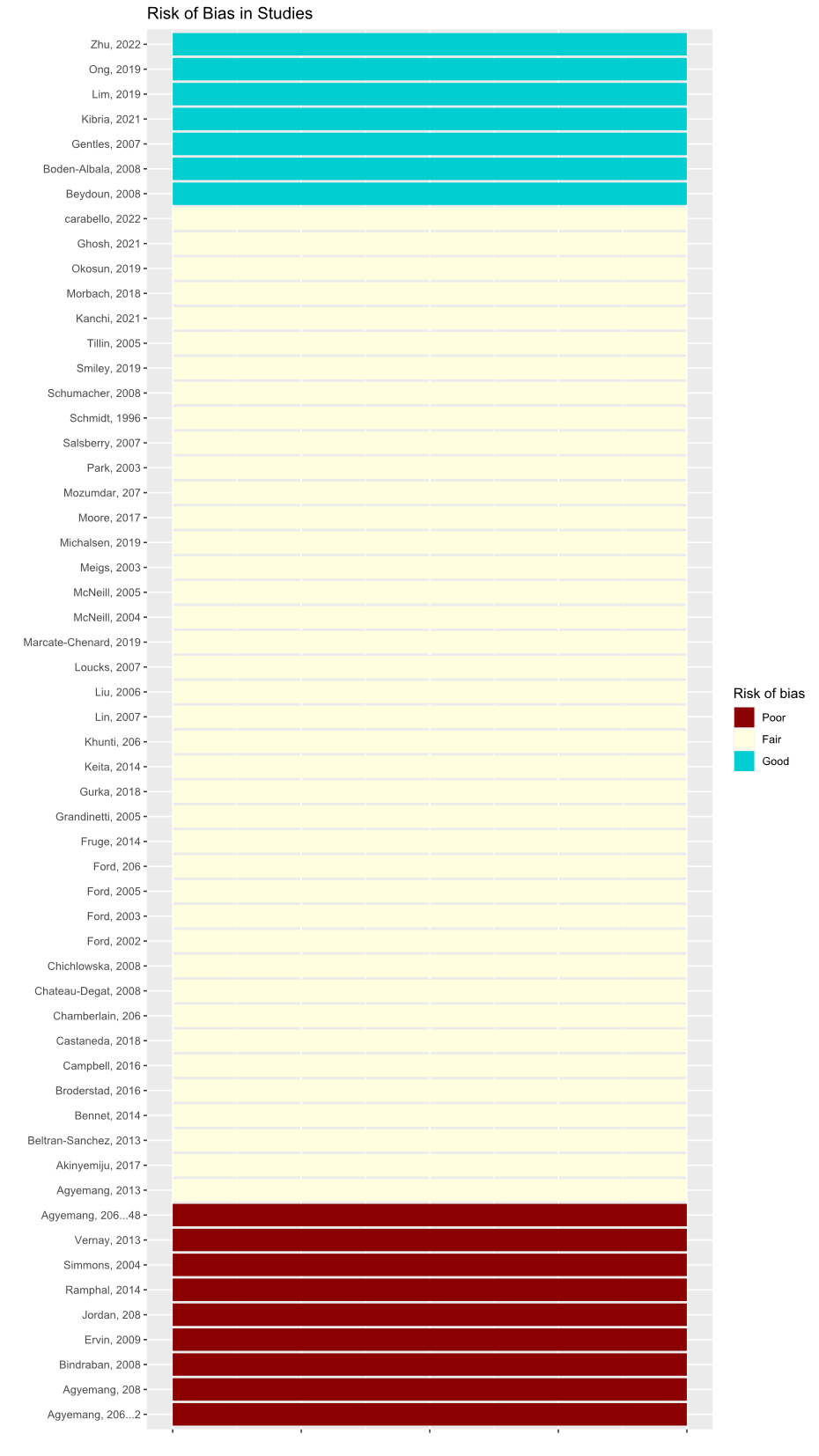
*

**Supplementary Table 3:** PRISMA Checklist

| **Section and Topic** | **Item #** | **Checklist item** | **Location where item is reported** |
| --- | --- | --- | --- |
| **TITLE** | | |  |
| Title | 1 | Identify the report as a systematic review. | Title page |
| **ABSTRACT** | | |  |
| Abstract | 2 | See the PRISMA 2020 for Abstracts checklist. | Abstract |
| **INTRODUCTION** | | |  |
| Rationale | 3 | Describe the rationale for the review in the context of existing knowledge. | Introduction,  Page 3, line 118-124 |
| Objectives | 4 | Provide an explicit statement of the objective(s) or question(s) the review addresses. | Introduction,  Page 3, line 122-124 |
| **METHODS** | | |  |
| Eligibility criteria | 5 | Specify the inclusion and exclusion criteria for the review and how studies were grouped for the syntheses. | Method  Page 4, line 151-155 |
| Information sources | 6 | Specify all databases, registers, websites, organisations, reference lists and other sources searched or consulted to identify studies. Specify the date when each source was last searched or consulted. | Method  Page 3, line 139-148 |
| Search strategy | 7 | Present the full search strategies for all databases, registers and websites, including any filters and limits used. | Supplementary, Table 1 |
| Selection process | 8 | Specify the methods used to decide whether a study met the inclusion criteria of the review, including how many reviewers screened each record and each report retrieved, whether they worked independently, and if applicable, details of automation tools used in the process. | Method  Page 4, line 157-167 |
| Data collection process | 9 | Specify the methods used to collect data from reports, including how many reviewers collected data from each report, whether they worked independently, any processes for obtaining or confirming data from study investigators, and if applicable, details of automation tools used in the process. | Method  Page 3, line 146-148 |
| Data items | 10a | List and define all outcomes for which data were sought. Specify whether all results that were compatible with each outcome domain in each study were sought (e.g. for all measures, time points, analyses), and if not, the methods used to decide which results to collect. | Method  Page 5, line 191-193 |
|  | 10b | List and define all other variables for which data were sought (e.g. participant and intervention characteristics, funding sources). Describe any assumptions made about any missing or unclear information. | Method  Page 5, line 191-192 |
| Study risk of bias assessment | 11 | Specify the methods used to assess risk of bias in the included studies, including details of the tool(s) used, how many reviewers assessed each study and whether they worked independently, and if applicable, details of automation tools used in the process. | Method  Page 4, line 181-188 |
| Effect measures | 12 | Specify for each outcome the effect measure(s) (e.g. risk ratio, mean difference) used in the synthesis or presentation of results. | Method  Page 4, line 200-207 |
| Synthesis methods | 13a | Describe the processes used to decide which studies were eligible for each synthesis (e.g. tabulating the study intervention characteristics and comparing against the planned groups for each synthesis (item #5)). | Method  Page 5, line 196--197 |
|  | 13b | Describe any methods required to prepare the data for presentation or synthesis, such as handling of missing summary statistics, or data conversions. | Method  Page 5, line 199--204 |
|  | 13c | Describe any methods used to tabulate or visually display results of individual studies and syntheses. | Method  Page 6, line 219--221 |
|  | 13d | Describe any methods used to synthesize results and provide a rationale for the choice(s). If meta-analysis was performed, describe the model(s), method(s) to identify the presence and extent of statistical heterogeneity, and software package(s) used. | Method  Page 5, line 208--212 |
|  | 13e | Describe any methods used to explore possible causes of heterogeneity among study results (e.g. subgroup analysis, meta-regression). | Method  Page 5, line 213--216 |
|  | 13f | Describe any sensitivity analyses conducted to assess robustness of the synthesized results. | Method  Page 5, line 216--218 |
| Reporting bias assessment | 14 | Describe any methods used to assess risk of bias due to missing results in a synthesis (arising from reporting biases). | Method  Page 5, line 211--212 |
| Certainty assessment | 15 | Describe any methods used to assess certainty (or confidence) in the body of evidence for an outcome. | Method  Page 5, line 202--204 |
| **RESULTS** | | |  |
| Study selection | 16a | Describe the results of the search and selection process, from the number of records identified in the search to the number of studies included in the review, ideally using a flow diagram. | Results  Page 6, line 223-226 |
|  | 16b | Cite studies that might appear to meet the inclusion criteria, but which were excluded, and explain why they were excluded. | Figure 1 |
| Study characteristics | 17 | Cite each included study and present its characteristics. | Table 1 |
| Risk of bias in studies | 18 | Present assessments of risk of bias for each included study. | Results  Page 7, line 262-263 |
| Results of individual studies | 19 | For all outcomes, present, for each study: (a) summary statistics for each group (where appropriate) and (b) an effect estimate and its precision (e.g. confidence/credible interval), ideally using structured tables or plots. | Figure 2 - 4 |
| Results of syntheses | 20a | For each synthesis, briefly summarise the characteristics and risk of bias among contributing studies. | Results  Page 6, line 229-243 |
|  | 20b | Present results of all statistical syntheses conducted. If meta-analysis was done, present for each the summary estimate and its precision (e.g. confidence/credible interval) and measures of statistical heterogeneity. If comparing groups, describe the direction of the effect. | Results  Page 7, line 272-291 |
|  | 20c | Present results of all investigations of possible causes of heterogeneity among study results. | Results  Page 8, line 292-298 |
|  | 20d | Present results of all sensitivity analyses conducted to assess the robustness of the synthesized results. | Results  Page 8, line 300-304 |
| Reporting biases | 21 | Present assessments of risk of bias due to missing results (arising from reporting biases) for each synthesis assessed. | Results  Page 7, line 277-278 |
| Certainty of evidence | 22 | Present assessments of certainty (or confidence) in the body of evidence for each outcome assessed. | Results  Page 8, line 305-309 |
| **DISCUSSION** | | |  |
| Discussion | 23a | Provide a general interpretation of the results in the context of other evidence. | Discussion  Page 9, line 331-363 |
|  | 23b | Discuss any limitations of the evidence included in the review. | Discussion  Page 10, line 367-390 |
|  | 23c | Discuss any limitations of the review processes used. | Discussion  Page 10, line 377-388 |
|  | 23d | Discuss implications of the results for practice, policy, and future research. | Discussion  Page 11, line 398-409 |
| **OTHER INFORMATION** | | |  |
| Registration and protocol | 24a | Provide registration information for the review, including register name and registration number, or state that the review was not registered. | Abstract, line 76 |
|  | 24b | Indicate where the review protocol can be accessed, or state that a protocol was not prepared. | Page 3, line 128-129 |
|  | 24c | Describe and explain any amendments to information provided at registration or in the protocol. | N/a |
| Support | 25 | Describe sources of financial or non-financial support for the review, and the role of the funders or sponsors in the review. | Page 11, line 412 |
| Competing interests | 26 | Declare any competing interests of review authors. | Page 11, line 413 |
| Availability of data, code and other materials | 27 | Report which of the following are publicly available and where they can be found: template data collection forms; data extracted from included studies; data used for all analyses; analytic code; any other materials used in the review. | Page 11, line 410 |
